# 1464. β-lactam versus Non-β-lactam Alternatives for Colorectal Surgical Prophylaxis: Does Recommended Dosing and Timing Matter?

**DOI:** 10.1093/ofid/ofad500.1301

**Published:** 2023-11-27

**Authors:** Curtis D Collins, Eric Hartsfield, Robert K Cleary, Kara K Brockhaus

**Affiliations:** Trinity Health Ann Arbor, Ypsilanti, Michigan; Michigan Medicine, Ann Arbor, Michigan; Trinity Health Ann Arbor, Ypsilanti, Michigan; Trinity Health Ann Arbor, Ypsilanti, Michigan

## Abstract

**Background:**

Previous studies have shown reduced surgical site infections (SSIs) with β-lactam prophylaxis compared with non-β-lactam alternatives; however, variations in timing and dosing have been reported. Few studies have investigated the impact of preoperative antibiotic timing or dosing on reported SSI differences between regimens.

**Methods:**

A multi-center, retrospective cohort study was performed utilizing available data from elective colorectal surgeries in Michigan. Adult patients receiving surgical prophylaxis between July 2012 and June 2021 were included. Exclusions included emergent or urgent surgeries and missing follow-up and antibiotic information. The primary objective was to evaluate the incidence of SSIs between patients receiving surgical prophylaxis with β-lactam antibiotics with those who received non-β-lactam alternatives. A multivariable logistic regression model tested the association of SSIs and covariates including antibiotic dosing and timing.

**Results:**

A total of 28,899 procedures were included. Of these, 25,875 received β-lactam prophylaxis and 3,024 received non-β-lactam alternatives. Patients in the β-lactam cohort received higher recommended dosing (83.7% vs. 54.2%; p < 0.001), timing (93.6% vs. 83.9%; p < 0.001), combined recommended dose and timing (78.7% vs. 38.1%; p < 0.001), and had a lower unadjusted incidence of SSIs (6.7% vs 8.9%, p < 0.001). Multivariable logistic regression showed β-lactam prophylaxis was associated with a reduction in SSIs (adjusted odds ratio (aOR) 0.79; 95% CI 0.64 to 0.98; p =0.033) (Table 1). Recommended dosing (aOR 1.09; 95% CI 0.91 to 1.32; p = 0.359) and timing (aOR 1.07; 95% CI 0.83 to 1.39; p = 0.601) were not associated with any change in SSIs. Older age clean/contaminated procedures, and colectomies were associated with a decreased SSI risk. Higher body mass index, corticosteroid use, contaminated and dirty procedures, ASA scores 3-5, and open procedures were associated with an increased risk.

**Figure:**
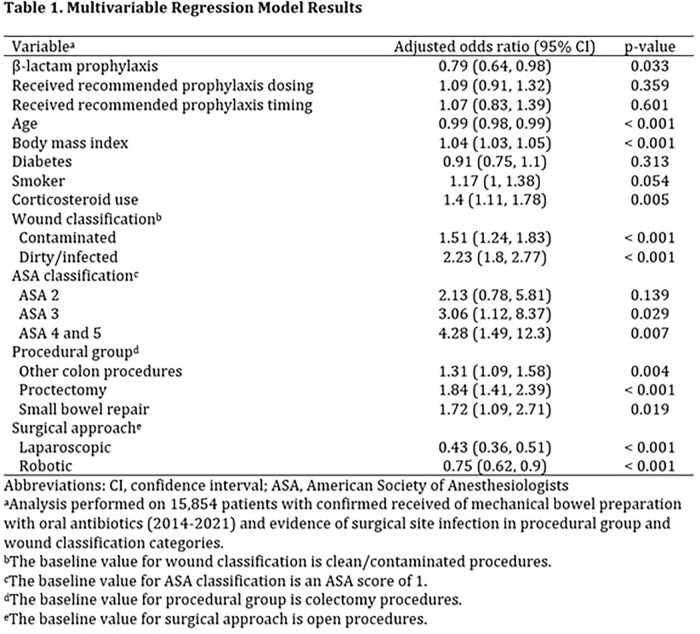

**Conclusion:**

The use of β-lactams for surgical prophylaxis in elective colorectal surgery procedures was associated with decreased SSIs. Significant differences in compliance with recommended prophylaxis dosing and timing between cohorts was not associated with changes in SSIs.

**Disclosures:**

**Robert K. Cleary, MD, FASCRS, FACS**, Intuitive Surgical Inc: Honoraria

